# The impact of empagliflozin and metformin on cardiac parameters in patients with mid-range ejection fraction heart failure without diabetes

**DOI:** 10.25122/jml-2023-0340

**Published:** 2024-01

**Authors:** Reeman Sabbar, Sinaa Abdul Amir Kadhim, Hayder Adnan Fawzi, Ali Flayih, Bassim Mohammad, Asma Swadi

**Affiliations:** 1 Department of Pharmacology, College of Medicine, University of Al-Qadisiyah, Al-Qadisiyah, Iraq; 2 Department of Pharmacy, Al-Mustafa University College, Baghdad, Iraq

**Keywords:** heart failure, sodium-glucose transport protein 2 (SGLT2) inhibitors, metformin, echocardiography, N-terminal pro-BNP, FFA, Free Fatty Acid, HFmrEF, Heart Failure with mid-range Ejection Fraction, LVEDD, Left Ventricular End-Diastolic Diameter

## Abstract

Heart failure (HF) remains a significant problem for healthcare systems, requiring the use of intervention and multimodal management strategies. We aimed to assess the short-term effect of empagliflozin (EMPA) and metformin on cardiac function parameters, including ventricular dimension-hypertrophy, septal thickness, ejection fraction (EF), and N-terminal pro-brain natriuretic peptide (NT-proBNP) levels in patients with HF and mildly reduced EF. A case-control study included 60 newly diagnosed patients with HF. Patients were divided into two groups: Group E received standard HF treatment (carvedilol, bumetanide, sacubitril-valsartan, spironolactone) plus EMPA 10 mg daily, and Group M received standard HF treatment plus metformin 500 mg daily. After three months of treatment, Group E had a significantly higher EF than Group M compared to initial measurements (a change of 9.2% versus 6.1%, respectively). We found similar results in the left ventricular end-systolic dimension (LVESD), with mean reductions of 0.72 mm for Group E and 0.23 mm for Group M. Regarding cardiac indicators, the level of NT-proBNP was considerably decreased in both groups. However, the reduction was significantly greater in group E than in group M compared to the initial level (mean reduction: 719.9 vs. 973.6, respectively). When combined with quadruple anti-heart failure therapy, metformin enhanced several echocardiographic parameters, showing effects similar to those of EMPA when used in the same treatment regimen. However, the benefits of EMPA were more pronounced, particularly regarding improvements in EF and LVESD.

## INTRODUCTION

Heart failure (HF) is a medical condition in which the heart does not function efficiently, resulting in symptoms such as difficulty breathing, swelling in the ankles, and fatigue. These symptoms may be accompanied by physical signs like increased pressure in the jugular veins, crackling sounds in the lungs, and swelling in the extremities. This condition is characterized by abnormalities in the structure or function of the heart, which leads to a decrease in the amount of blood pumped by the heart or an increase in the pressure within the heart, either at rest or during times of stress [1-4]. Heart failure has a worldwide impact, affecting around 64.3 million people [5]. Approximately 1–2% of the total population in countries with higher economic status have received a diagnosis of heart failure [6].

The treatment of HF involves several classes of drugs, including beta blockers, loop diuretics, angiotensin-converting enzyme inhibitors (ACEi), angiotensin receptor blockers (ARB), and aldosterone antagonists, among others [7]. The combination of different drug classes has led to improved survival rates in heart failure treatment, making such integrated approaches standard practice [7,8].

Empagliflozin (EMPA), initially approved for type 2 diabetes mellitus (T2DM), offers versatility as it can be prescribed both as a standalone treatment for diabetes or in combination with other medications [9,10]. Beyond its primary indication, EMPA has also been recognized for its efficacy in treating (HF) among patients with and without DM, as evidenced by clinical trials showing improved survival rates [9]. EMPA has been effective in reducing hospitalizations due to heart failure and in reducing mortality caused by cardiovascular (CV) events. Patients with T2DM carry an increased risk of CV mortality [11,12]. EMPA works by blocking the sodium-glucose cotransporter-2 (SGLT-2) protein in the kidneys, which normally helps reabsorb glucose from the urine back into the bloodstream. By inhibiting this protein, EMPA reduces glucose reabsorption, excreting more glucose in the urine. Beyond its effects on glucose levels, EMPA also aids in weight loss and reduces blood pressure without affecting heart rate [13].

The release of results from the EMPA-REG OUTCOME trial marked a milestone, as EMPA became the first medication proven to reduce mortality from cardiovascular causes in patients with T2DM [14]. EMPA has glucuronic, diuretic, and natriuretic characteristics, lowering blood glucose, inducing osmotic diuresis, and lowering sodium burden, respectively [15]. This makes EMPA a valuable addition to the standard treatments of patients with T2DM and pre-existing cardiovascular disease by reducing cardiovascular events, cardiovascular mortality, and the incidence or progression of nephropathy; it also significantly reduces myocardial fibrosis [14]. Empagliflozin also offers cardiovascular benefits to patients without diabetes. Its effectiveness in reducing hospitalizations and mortality rates has established EMPA as a cornerstone in managing heart failure with reduced ejection fraction (HFrEF). Additionally, patients with heart failure with preserved ejection fraction (HFpEF) also benefited from EMPA treatment, irrespective of concurrent diabetes status [16].

Metformin, while primarily known as an effective anti-diabetic medication, also has certain benefits for individuals with heart failure [17,18]. The heart derives most of its energy by utilizing free fatty acid (FFA) through an oxidation process. The rest of the energy comes from glucose and lactic acid metabolism [19]. Metformin is believed to exert its effects by enhancing mitochondrial FFA oxidation, which reduces the heart's production of advanced glycation end-products and minimizes cardiac cell apoptosis by activating adenosine monophosphate-activated protein kinase [20]. Two randomized clinical trials (RCTs) examined the mechanisms of metformin in heart failure treatment. In the first trial, individuals with insulin resistance and HFrEF received either metformin or placebo over four months. While maximal oxygen consumption remained unchanged, cardiac contraction efficiency improved [21]. The second trial demonstrated that metformin enhanced cardiac cell function, as indicated by an increase in the cardiac metabolic index and a reduction in the oxygen consumption of cardiac cells compared to the placebo group. This increase in the cardiac metabolic index was correlated with higher metformin levels [22]. The objective of this research was to assess the effects of EMPA and metformin on cardiac echocardiographic and biochemical indicators.

## MATERIAL AND METHODS

### Study design and setting

This prospective case-control study was conducted at Al-Diwaniyah Teaching Hospital, Iraq, between the 1^st^ of May 2022 and the 1^st^ of November 2022. We included 60 patients between 50 and 70 years diagnosed with HFmrEF, with an EF between 41.0% and 49.0% [23]. Patient data were retrieved from the inpatient ward files. The study protocol adhered to the Strengthening the Reporting of Observational Studies in Epidemiology (STROBE) Statement standard checklist [24].

### Participants

We included patients 70 years old or younger without diabetes who were recently diagnosed with Stage II and III heart failure, characterized by a mid-range ejection fraction. Patients were excluded if they had kidney dysfunction, treatment intolerance, or concomitant cardiac or brain disease other than HF. Other exclusion criteria were contraindication to metformin and EMPA use, thyroid disorders, and systolic blood pressure ≥180.0 mmHg.

Patients were divided into two groups:

**Group E:** 30 patients received standard-of-care quadruple treatment for heart failure with EMPA 10 mg once daily

**Group M:** 30 patients received standard-of-care treatment for heart failure with metformin 500 mg daily.

For all participants, key variables such as echocardiographic data and cardiac biomarkers were systematically collected at baseline - the point of treatment initiation - and again at the three-month follow-up to assess changes and treatment efficacy.

### Standard of care therapy

All patients received a standard heart failure regimen: 3,125 mg carvedilol daily, 1 mg bumetanide once or twice daily, 200 mg sacubitril-valsartan once daily, and 25 mg spironolactone twice daily. Group M received an additional 500 mg of metformin with the evening meal, while Group E received 10 mg EMPA in the morning, regardless of food consumption. All therapies adhered to the most up-to-date recommendations for the treatment of heart failure [23].

### Sample size

The determination of the sample size was conducted using the following formula:


$$\min imum\;sample\;size\;(n)=p\frac{\left(1-p\right)Z_{0.95}^{2}}{d^{2}}$$

The lowest sample size, denoted as *n*, was determined based on the prevalence of heart failure (2%), as reported in the Groenewegen *et al*. study [6,25], leading to 30 patients per group.

### Sample collection and laboratory analysis

We collected 10 ml venous blood samples from each participant, which was allowed to coagulate. The samples were then centrifuged at a rate of 2,000 to 3,000 revolutions per minute for 20 minutes to separate the supernatant for analysis. The N-terminal pro-brain natriuretic peptide (NT-proBNP) levels were determined using ELISA (Sunlong Biotech). This method uses antibodies reactive to the BNP antigen, and the optical absorbance is converted to concentration using a predefined standard curve. The levels of hemoglobin A1C (HbA1c) were determined using an enzymatic assay (Linear), which selectively measures the N-terminal fructose dipeptides of the glycated hemoglobin side chain [26].

### Echocardiography

Echocardiographic assessments, including EF, left ventricular end-diastolic diameter (LVEDD), left ventricular end-systolic diameter (LVESD), and interventricular septal thickness (IVST), were performed by a consultant cardiologist using a Vinno G60 (serial number 4011640003).

### Statistical analysis

The analysis was conducted using GraphPad Prism version 10.0. The Anderson–Darling test confirmed the normal distribution of variables. Discrete variables were presented as numbers and percentages and analyzed using the Chi-square test. Continuous variables were analyzed using paired and independent t-tests, with a *P* value of ≤0.05 indicating statistical significance.

## RESULTS

The study included 60 patients equally divided into two groups, each with 30 patients. There were no significant differences in the mean age, gender, body mass index (BMI), and HbA1c between both groups at baseline, as illustrated by Table 1.

**Table 1:** Patient characteristics at baseline

Variables	Group M (*n* = 30)	Group E (*n* = 30)	*P* value
**Age (years)**	59.2 ± 6.0	61.4 ± 5.7	0.153
**Gender, *n* (%)**			
**Women**	14 (46.7%)	7 (23.3%)	0.058
**Men**	16 (53.3%)	23 (76.7%)	
**BMI (kg/m^2^)**	26.1 ± 2.9	27.4 ± 2.4	0.066
**HbA1c (%)**	4.99 ± 0.32	4.98 ± 0.31	0.903

Data presented as mean ± standard deviation

**Figure 1:**
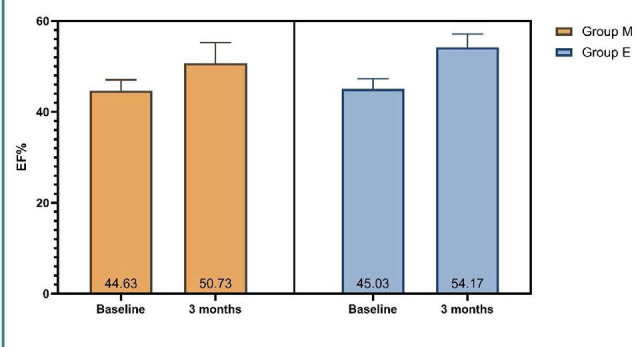
Ejection fraction across groups

**Figure 2:**
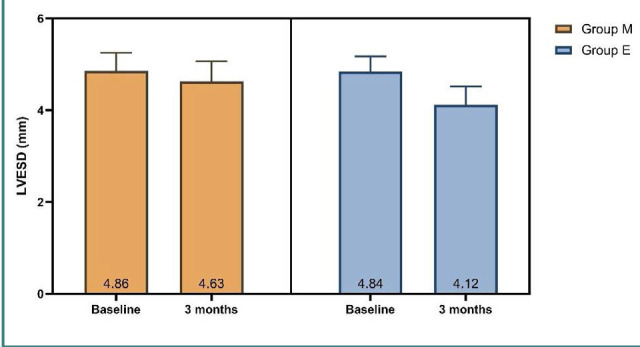
Left ventricular end-systolic diameter across groups

Ejection fraction significantly increased after three months of treatment in patients who received EMPA compared to those who received metformin and standard-of-care treatment for heart failure, with mean changes of 9.2 versus 6.1%, respectively (Figure 1). LVESD was significantly reduced in patients who received EMPA compared to those who received metformin in addition to standard-of-care treatment for heart failure (mean change 0.72, vs. 0.23 mm, respectively) (Figure 2). However, there were no significant differences after three months of treatment for LVEDD and IVST, as illustrated by Table 2.

**Table 2:** Assessment of echocardiographic variables

Variables	Group M (*n* = 30)	Group E (*n* = 30)	*P* value
**Ejection Fraction (%)**			
**Baseline**	44.6 ± 2.4	45.0 ± 2.2	0.504
**After three months**	50.7 ± 4.6	54.2 ± 3.0	0.001 [S]
***P* value**	<0.001 [S]	<0.001 [S]	
**LVEDD (mm)**			
**Baseline**	6.55 ± 0.36	6.53 ± 0.35	0.801
**After three months**	6.27 ± 0.34	6.15 ± 0.49	0.302
***P* value**	<0.001 [S]	<0.001 [S]	
**LVESD (mm)**			
**Baseline**	4.86 ± 0.39	4.84 ± 0.33	0.860
**After three months**	4.63 ± 0.44	4.12 ± 0.40	<0.001 [S]
***P* value**	<0.001 [S]	<0.001 [S]	
**IVS thickness (mm)**			
**Baseline**	0.86 ± 0.17	0.82 ± 0.17	0.330
**After three months**	0.83 ± 0.17	0.79 ± 0.17	0.328
***P* value**	0.001 [S]	<0.001 [S]	

Data presented as mean ± standard deviation

**Figure 3:**
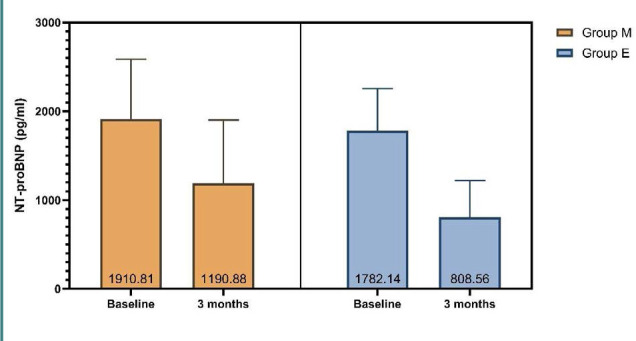
Assessment of cardiac NT-proBNP across study groups

Serum level of cardiac NT-proBNP was significantly reduced for both groups, and this reduction was significantly higher in patients who received EMPA than those who received metformin (mean reduction: 719.9 vs. 973.6, respectively), as illustrated by Table 3 and Figure 3.

**Table 3:** Assessment of cardiac NT-proBNP level

NT-proBNP	Group M (*n* = 30)	Group E (*n* = 30)	*P* value
**Baseline**	1,910.8 ± 673.7	1,782.1 ± 473.8	0.396
**After three months**	1,190.9 ± 711.0	808.6 ± 412.2	0.014
**Mean reduction**	719.9	973.6	
***P* value**	<0.001	<0.001	

Data presented as mean ± standard deviation

## DISCUSSION

In this study, all patients were administered a comprehensive therapy regimen for HF based on the most up-to-date guidelines [23]. Treatment with EMPA resulted in a significantly higher improvement of EF than what was observed with metformin therapy when both were combined with the standard of care. Moreover, EMPA also improved LVESD, indicating potential early cardiac remodeling benefits.

Given the scarcity of prospective randomized controlled trials in patients with HFmrEF, our findings are particularly unique since they showed that EMPA use in patients with HFmrEF and without diabetes can improve EF and key cardiac biomarkers, which suggests a survival benefit for the patients. This is our second study, which examined patients with HFmrEF without diabetes [27], and we initially presented evidence of these therapeutic benefits.

The current understanding of SGLT2 inhibitors (SGL2i) in heart failure with mid-range ejection fraction is primarily derived from retrospective analyses or subgroup assessments within broader heart failure trials that predominantly involve individuals with heart failure with reduced ejection fraction. Patients with HFmrEF seem to respond to medical therapy similarly to those with HFrEF. As a result, it might make sense to treat these patients with the same guideline-directed medical therapy employed in managing HFrEF, in which SGL2i is graded as level 2a according to the recent 2022 AHA/ACC/HFSA guidelines [23]. In an RCT that examined the effect of 10 mg EMPA versus placebo, EMPA showed significant improvement in EF after six months of treatment (6.0 vs. -0.1, *P* <0.001) [28]. There was a significant reduction in left ventricle end-diastolic volume (-25.1 vs. -1.5 ml, *P* <0.001) and left ventricle end-diastolic volume (-26.6 vs. -0.5, *P* <0.001), and left ventricle mass (-17.8 vs. 4.1 g, *P* < 0.001). These results align with the findings of our current study, suggesting that EMPA is effective in reducing cardiac remodeling, as evidenced by the improved left ventricular parameters and ejection fraction. Left ventricle reverse remodeling is a key component in decreasing the immediate and long-term mortality and morbidity in patients with HF [29,30,31]. The current findings, which demonstrate a reversal of cardiac remodeling in HF patients, support the outcomes reported by major RCTs, namely, EMPA-TROPISM, DAPA-HF, and EMPEROR-Reduced [28,32,33].

In the study by Hao *et al*., patients with HFrEF were administered 10 mg of EMPA daily for three months. At the end of therapy, there was a significant improvement in EF by 6.1%, significant reductions in LVEDD by 5.4 mm, and BNP by 1213.7 pg/ml, similar to our study. One major limitation in generalizing the findings of this study is the lack of comparison with a placebo group. However, their findings align with the current study [34]. Conversely, other studies reported conflicting findings, where EMPA treatment in HFrEF patients over 12 weeks did not result in statistically significant improvements in EF compared to placebo, despite a higher EF valued in the EMPA group (mean change from baseline 2.4 vs. 1.0 between EMPA vs. placebo, *P* = 0.32). This discrepancy could be attributed to the short duration of treatment. In addition, LVESV and LVEDV were reduced in the EMPA group compared to placebo [35]. Patients in the EMPEROR Reduced trial were diagnosed with HFrEF, while our study included patients with HFmrEF, which could explain the earlier benefits of EMPA in the present study compared to the EMPEROR Reduced trial.

LVEDD is an effective echocardiographic indicator for evaluating cardiac chamber size and diastolic function, making it particularly useful in patients undergoing myocardial remodeling or presenting with abnormal cardiac structures. A study examining patients with heart failure found that LVEDD and LVESD increments can predict changes in LVEF in patients with heart failure and an LVEF < 35% [36]. In the current study, LVEDD and LVESD were significantly reduced after EMPA treatment. To our knowledge, no previous study reported this finding in the literature.

Innovative anti-diabetic drugs SGLT2 improve glycemic control without increasing insulin production. The ability of SGLT2 to lower blood sugar levels alone cannot account for the CV improvements in heart failure. This contrasts with other traditional hypoglycemic drugs with comparable or greater anti-hyperglycemic capabilities, such as sulfonylureas and dipeptidyl peptidase inhibitors, for patients with HF [37]. SGLT2 inhibitors operate by inducing osmotic diuresis, effectively blocking the reabsorption of salt and glucose [38]. The natriuretic activity of SGLT2 is linked to a larger fluid decrease in the interstitial compartment than in the intravascular compartment when compared to loop diuretics. Consequently, congestion is reduced, with little to no impact on effective circulation volume or organ perfusion [39]. Additionally, it appears that the slight impact on plasma volume reduces both preload and afterload, which aids in reversing cardiac remodeling [40] without increasing sympathetic nerve activity [41]. However, research has demonstrated that the diuretic impact of SGLT2 wears off very quickly. Some studies have found no association between volume status and the benefits of SGLT2I in patients with HFrEF [42]. Research involving animal models, including rabbits and rats, has shown that SGLT2 inhibitors can downregulate the cardiac sodium-hydrogen exchanger. This action leads to decreased levels of cytosolic sodium and calcium in the myocardium while increasing mitochondrial calcium concentration [43]. These results resulted in better cardiac hypertrophy, fibrosis, remodeling, enhanced mitochondrial function, oxidative stress, and cardiac contractile activity [44].

The prescribing guidelines for metformin in the treatment of heart failure have recently been updated to remove the previous contraindication. The modification was based on increasing evidence that supports the safety and benefits of metformin for individuals with diabetes and heart failure, gathered from clinical observations and experimental investigations [45]. In the past, the risk of lactic acidosis has deterred the prescription of metformin to patients with heart failure [46].

A meta-analysis has found that metformin is considered safe for patients with both diabetes mellitus and heart failure, irrespective of the presence of HFrEF or chronic kidney failure. On the other hand, there is no empirical data to indicate that metformin has a higher propensity for causing lactic acidosis compared to other drugs employed for reducing blood glucose levels [47].

Only a few studies have investigated the correlation between metformin and heart failure in individuals without diabetes [27]. One study focused on the effects of metformin over six months in patients with metabolic syndrome and found significant improvements in EF (*P* value <0.003) [48]. Additionally, another study examining the impact of metformin on patients with DM reported a significant reduction in BNP levels, indicating a 40% decrease compared to the control group [49]. Furthermore, a meta-analysis involving 754 non-diabetic patients with left ventricular hypertrophy highlighted the ability of metformin to enhance LVEF after a year of treatment [50]. Another study on HFrEF showed a modest 1% improvement in EF after three months of metformin therapy [51]. The results aligned with a study by Wong and colleagues, which investigated heart failure in individuals without diabetes. In this study, patients were given metformin for four months, and their outcomes were compared to those of a control group. They found slight improvements in EF in the metformin group, but these gains were not statistically significant compared to the control group [52]. The study conducted by Rosiak *et al*. revealed that the use of metformin was associated with lower levels of B-type BNP in individuals with T2DM, suggesting a negative correlation between metformin use and high BNP levels [53]. This study has several limitations, including its short duration and the lack of long-term morbidity and mortality outcomes.

## CONCLUSION

EMPA plays an important role in improving cardiac events by improving various echocardiographic parameters like EF, end-systolic/diastolic volume, and cardiac biomarkers like troponin. Metformin may produce a cardiac protective effect by reducing EF. Integrating metformin into the standard cardiac treatment regimen could provide additional benefits for patients with heart failure.
